# Healthcare utilisation for elderly people at the onset of the COVID-19 pandemic in South Korea

**DOI:** 10.1186/s12877-022-03085-5

**Published:** 2022-05-06

**Authors:** K. Park, J. Byeon, Y. Yang, H. Cho

**Affiliations:** Health Insurance Research Institute, National Health Insurance Service, Wonju, 26464 South Korea

## Abstract

**Background:**

At the onset of the coronavirus disease 2019 (COVID-19) pandemic, health care systems were severely disrupted in many countries and in particular, elderly people vulnerable to COVID-19 may have been reluctant to receive their medical treatment.

**Methods:**

We conducted interrupted time series analyses (ITSA) using nationwide medical claim data between January 2020 and July 2020, with focus on different disease categories for the patients of 65 to 84-year-olds, i.e., acute upper respiratory infections (AURIs) vs. chronic diseases.

**Results:**

AURIs and chronic diseases showed a sharp contrast with respect to the change in healthcare service utilisation. First, the utilisation rate for chronic diseases changed little whereas for AURIs it dropped by 20.4% year-over-year (yoy) at the onset of the pandemic (week 6, 2020). Second, as social distancing relaxed (week 17, 2020), the AURIs patients trended up and even reached to 7.8% above yoy whereas no significant change found for chronic diseases.

**Conclusions:**

The uninterrupted treatment for chronic diseases in contrast to the AURIs implies that the governmental and public responses to the pandemic outbreak worked for efficient healthcare provision to patients in needs of regular check-ups and treatment in the middle of an infectious disease crisis.

**Supplementary Information:**

The online version contains supplementary material available at 10.1186/s12877-022-03085-5.

##  Background

In many countries, there has been a considerable decline in health care use by non-coronavirus disease 2019 (COVID-19) patients [1–5]. Some health care facilities could not sustain the pre-pandemic level of services because their resources shifted towards the COVID-19 patients, and some facilities were shut down to avoid disease transmission. In addition, people were reluctant to visit medical institutions because of the fear of infection. Social distancing policies, such as stay-at-home orders, which limit the movement of people, also hindered medical facility utilisation. Given this situation, the use of essential medical services, not to mention elective services, decreased [6]; for example, by 38% for severe heart attack patients treated in nine major hospitals in the U.S. [7], 64% in paediatric emergency room visits in Germany [8], and 50% reductions in emergency departments visits in Italy [9].

Restricted access to health care facilities usually has the greatest impact on the most vulnerable groups. In particular, older people might be at higher risk of poor outcomes due to reduced or delayed healthcare services because they tend to have co-morbidities and chronic diseases associated with complications when timely care is not provided [6, 10, 11]. Elderly individuals with pre-existing chronic illnesses, such as cardiovascular disease, renal failure, cerebrovascular disease, respiratory disease, cancer, and diabetes, are more prone to the risk of mortality [12–14]. Moreover, elderly people with chronic diseases inevitably have greater fear of infection and are more likely to refrain from utilising medical care [15].

If patients with chronic diseases (or others in need of ongoing care) defer or delay hospital visits due to the fear of acquiring COVID-19 or the lack of hospital capacity, then this may have long-term negative effects on their health [16–18]. Moreover, the undesirable health outcomes may be more pronounced for older people. Patients with diabetes have a high risk of severe complications, including adult respiratory distress syndrome and multi-organ failure [19]. Previous studies also found that the management of hypertension and diabetes in elderly people is closely related to hospitalisation for related injuries, emergency room visits, and health care costs [20–22].

However, among the studies, which have explored healthcare utilisation during the COVID-19 pandemic, only few have focused on the healthcare utilisation for chronic diseases in elderly people. In the early stage of the COVID-19 pandemic, many research in healthcare utilisation has primarily focused on emergency admissions with urgent conditions (including severe heart attack or emergency visits). The COVID-19 pandemic is still ongoing, even with vaccines and medications. Therefore, our study is applicable and important in healthcare policy, emphasising the importance of providing medical services for chronic diseases with less indication of urgency but more severity in the long term for elderly patients. The negligence on chronic diseases might cause great public health loss even if it is not urgent or fatal.

We fill this gap in the existing research by using population-based healthcare claim data from the National Health Insurance Service of South Korea, collecting individual medical care information provided by the mandatory social health insurance system. We specifically investigate healthcare utilisation for chronic diseases in older patients before and during the COVID-19 pandemic, where most of the healthcare utilisation and cost is run by a national single-payer system.

South Korea is one of the most successful countries that showed an initial response to COVID-19 [23–26]. Because of the large-scale outbreak in Shincheonji, Daegu, starting on February 18, 2020, the number of newly confirmed cases per day peaked at 1062 on March 1, followed by a period of sustained transmission [27]. However, the daily average number of newly infected patients from March 15 to September 30 was 79.8 [28], and the number remained stable without travel restrictions or lockdown. In this context, South Korea can provide a good example for reviewing the changes in medical facility utilisation during the COVID-19 pandemic and the related policy effect. South Korea had relatively fewer barriers than other countries, wherein social distancing was mandated. Therefore, this study investigated the changes in healthcare utilisation for the older population of South Korea using nationwide data for individuals with different types of diseases to examine the unexpected endemic event on healthcare utilisation. The different stages of the pandemic are expected to reflect the extent of COVID-19 spread and follow the stages of control measures in South Korea.

## Methods

### Datasets

This study utilised South Korea’s National Health Insurance (NHI) claims data. Because this dataset includes data of the entire national population and all medical institutions are designated as providers, the NHI claims database represents the population’s utilisation of medical care and has been used to estimate the prevalence and incidence of diseases in South Korea.

This study targeted data from January 2020 to July 2020, and for comparison, a dataset containing data from January 2017 to December 2019 was constructed. It takes a certain amount of time for a hospital’s claims to be registered in the NHI data system, and the larger hospitals tend to claim multiple cases at once. Therefore, there is a delay between the actual date of medical care and the date in the system that indicates utilisation. To address this problem, we extracted the data after 6 months of last hospital visits (when the care was received on July 31, 2020, and the data was extracted on December 18, 2020) since almost 95% of claims are reported within 6 months [29].

### Elderly group classification

With high life expectancy of the South Koreans, similar to developed countries, the elderly in their 60s and 70s are quite active, fit, and able to care for themselves [30]. This observation corresponds to the two relatively active elderly sub-groups defined by gerontologists, i.e., young-old (65–74 years) and middle-old (75–84 years) [31]. Indeed, as of 2020, 4 out of 10 South Korean senior citizens from the sub-group of very old (≥85 years) live and stay in senior care centres or nursing homes, while being served not only casual maintenance but also most medical treatments from in-house medical staffs. Consequently, an inclusion of the very olds into our analysis creates statistical noises in two respects: First, any regular treatments carried out within senior care centres and nursing homes often constitute a part of contract and do not count treatment-by-treatment cases. Second, since their outings have to be approved by care home medical staffs and/or are often helped and guided by a family member or a helper, their hospital visits tend to be heavily affected by others’ circumstances, which contaminate the causality between social distancing policy and patient’s own decisions. For this statistical reasoning and to minimise related biases, we confine the elderly group to those aged 65–84 years for our main analysis.^1^

### Disease categories

For patients who visited the medical institutions taking ambulatory services during those periods, we extracted the claims with acute upper respiratory infections (AURIs), cancer, diabetes, or hypertension as the main and first sub-diagnosis. We identified the patient visit records pertaining to AURIs according to the code J00-J06 from the International Statistical Classification of Diseases and Related Health Problems (ICD). We considered diabetes (ICD-10 codes: E10, E11, E12, E13, E14) and hypertension (ICD-10 codes: I10, I11, I12, I13, I14, I15) as two representative chronic diseases. Finally, we used the data regarding cancer (denoted as “C” in the ICD-10 codes).

### Treated and control groups

We chose AURIs as the treatment group for an interrupted time series analysis (ITSA) because respiratory diseases have similar symptoms as those of COVID-19, and the level of “infectiousness” is usually inferred based on the amount of viral shedding from the upper respiratory tract in patients [32]. The fear and uncertainty around COVID-19 might have led people to refrain from physical and social interaction, which would help to reduce the spread of AURIs. Moreover, the anxiety of visiting a hospital for a minor illness might have kept the elderly population from seeking hospital services. Therefore, a substantial reduction in the incidence of AURIs could have implications for COVID-19 responses [33].

However, the incidence of chronic disease is independent of the COVID-19 pandemic and social distancing measures. Furthermore, individuals with chronic diseases visit the hospital regularly for prescriptions and check-ups. Healthcare utilisation according to chronic disease type can indicate whether hospital visits from elderly people have been affected by the COVID-19 pandemic due to the fear of infection or social distancing. In addition, there might be decreases in treatments because mandatory health check-ups have been delayed or deferred. Considering this, our results might underestimate the hospital visits associated with chronic diseases. The medical needs of patients with cancer might not be affected by the COVID-19 pandemic, but because of the fear of acquiring COVID-19, patients may avoid in-person visits to the hospital and postpone their appointments [34].

### Empirical methodology

#### ITSA (Interrupted Timeseries Analysis)

Randomised controlled trials (RCTs) are regarded as an essential design for evaluating the effectiveness of an intervention, but less suitable for the population level intermediation [35–37]. With this caveat of RCTs, ITSA design is well-fitting for evaluating the overall public health impacts of the COVID-19 pandemic on the people. Therefore, it has been increasingly used in the studies of the interventions of public health policies, predominantly the population level study without controls [38–40].

#### Single ITSA

We set two interventions; the first intervention time as the first week of February 2020 (week 6, 2020), when the first COVID-19 case in South Korea was reported in week 5, 2020; because of the high fear of acquiring it and uncertainty on COVID-19 in South Korea, there was a shortage of N95 masks and hand sanitisers; the second intervention time was set at the fourth week of April 2020 (week 17, 2020), when the level of social distancing was relaxed after the number of new COVID-19 cases decreased to less than 20 cases. We performed a single-group ITSA using Newey-West robust errors with one lag on the year-over-year (yoy) growth rate in the number of patients per week related to each disease as the dependent variable.

#### Outcome measures

Outcome measures of interest in this study are the yoy growth rate in the number of patients per week. To begin with, we examine the level change of the yoy growth rate by two interventions. Furthermore, we focus on the trend change of the yoy growth rate as well as the difference of post-intervention trend change of the yoy growth rate between the treated and control groups in multiple group ITSA, in order to estimate the intervention effect after implementation to the counterfactuals.

#### Multiple group ITSA

We extended a single ITSA to a multiple group ITSA, including the control group. We compared the interruption effect of hospital visits from AURIs and other diseases (hypertension, diabetes, cancer, and total ambulatory services). First, we defined the treatment group as those with AURIs because they have a similar transmission mechanism as that of COVID-19, and the patients might be affected by the widespread behavioural changes related to the pandemic. Second, we identified the control group as those with chronic diseases because their incidence has little association with the COVID-19 pandemic, but routine medical care is essential. Finally, we added cancer outpatient visits as the control group because their appointments should not be deterred but might be deferred due to the fear of infection during hospital visits. In multiple group ITSA, we used the data from 2017 to 2020, since the trend and the difference in the level before the intervention should be similar among treated and control groups.

### Statistical tools

Data extraction was performed using SAS software (ver. 9.4). We conducted our ITSA analysis by utilizing a publicly available package, which is written to run on STATA [41].

### Summary statistics

Table 1 presents the summary statistics for hospital visits by patients aged 65–84 years from January to July 2017–2020. Panel (a) shows the summary statistics for the period of January 1, 2020 to July 31, 2020, which represents 1) the number of patients per week 2) the yoy growth in the number of patients per week for those aged 65–84 years at the onset of the COVID-19 pandemic crisis. Panel (b) shows the summary statistics between January 1 and July 31, 2017, 2018, and 2019. Patients’ visits for total ambulatory services and chronic diseases did not change by much, while the number of patients with AURIs decreased during the COVID-19 pandemic by 18.7% (with a standard deviation of 0.313) between the same week in 2019 and 2020. On the other hand, the number of patients with most diseases increased from 2017 to 2019.

**Table 1:** Summary statistics for hospital visits for ambulatory services by 65–84-year-olds before and during the pandemic (2017 ~ 2019)

Diseases		(a) January 1st-July 31st, 2020		(b) January 1st-July 31st (2017, 2018, 2019)	
		Mean	Standard Dev	Mean	Standard Dev
**Total**	Number of patients per wk.	5,128,322	1,103,284	5,201,306	1,010,169
	Change in the number of patients per wk.	0.031	0.278	0.05	0.313
**Hypertension**	Number of patients per wk.	513,163	22,902	542,579	40,086
	Change in the number of patients per wk.	−0.029	0.156	−0.007	0.108
**Diabetes**	Number of patients per wk.	767,402	34,972	718,213	59,682
	Change in the number of patients per wk.	0.033	0.164	0.054	0.116
**Cancer**	Number of patients per wk.	75,990	6133	54,665	10,238
	Change in the number of patients per wk.	0.178	0.24	0.221	0.191
**AURIs**	Number of patients per wk.	111,645	54,308	135,613	38,837
	Change in the number of patients per wk.	−0.187	0.313	0.03	0.158

*Note*: This table presents the summary statistics for hospital visits for ambulatory services by 65–84-year-olds. Panel(a) covers the period between January 1st and July 31st in 2020 while panel (b) the same period of panel (a) in year 2017, 2018, and 2019. Mean represents the average number of weekly patients from each disease. The change in the volume of patients is calculated as the difference between the same week from the previous year (i.e., (week 6(2020)-week6(2019)]/week6(2019)). The number of patients only include ones taking ambulatory services

## Results

### Single ITSA

**Fig. 1:**
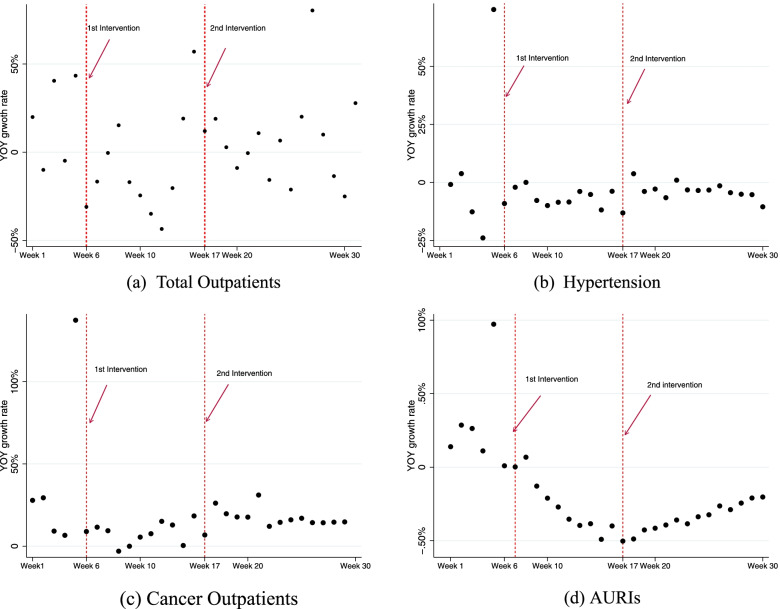
Presentation of single ITSA with two interventions for total, hypertension, cancer, and AURIs. *Note*: Fig. 1 represents the single interrupted time series analysis with two interventions. The outcome measure is the yoy growth rate for the number of patients from each disease per week between January–July, 2019 and 2020. The first intervention is week 6, 2020 and the second one week 17, 2020

Figure 1 shows the effect of the COVID-19 pandemic on older people’s hospital visits for total ambulatory services (A), hypertension (B), cancer (C), and AURIs (D). In contrast to the healthcare utilisation due to AURIs, in which AURI cases decreased compared to that in the previous year, other healthcare utilisation among patients aged 65–84 years did not change by much.

The relationship between healthcare utilisation before and during the COVID-19 pandemic showed no specific trend for most disease categories. After the COVID-19 pandemic began, the yoy of AURIs showed a clearly negative trend, and a gradual upward slope after the social distancing measure was relaxed. However, in hypertension and cancer outpatients, the yoy did not show a distinct trend, but later it showed a slight upward slope. The distinctive correlation between two intervention periods validates ITSA rather than a linear regression for further analysis.

Table 2 shows the results of a single ITSA with AURIs and other diseases. The level and trend change of the yoy growth rate after the first intervention have negative values and a statistically significant difference only in the AURIs, while visits for chronic diseases tended to be negatively associated with the intervention but without any statistical significance. Once the interruption starts, the patient visits due to AURIs drop by 75% (*p* < 0.05); moreover, the post-interruption trend shows an ongoing downward slope of 20.4% (*p* < 0.01). The second interruption event was set when mandated social distancing measures were relaxed, for example, after school opening. The post-interruption trend reverses in cases of AURIs by 7.8% (*p* < 0.001), although the healthcare utilisation for other diseases had little change from that of the previous year. This indicates that relaxing the social distancing had an increasing effect on hospital visits for patients diagnosed with AURIs, while hospital visits for patients diagnosed with chronic diseases were not affected.

**Table 2:** Estimated results for multiple group ITSA with two interventions for total, chronic diseases, cancer, and AURIs

		AURIs	Total	Hypertension	Diabetes	Cancer
Intercept	Coefficients	0.056	0.074	−0.164	−0.116	0.028
	95% CI	−0.162,0.275	−0.102,0.250	−0.491,0.163	−0.460,0.228	−0.448,0.504
	*P-value*	0.599	0.396	0.310	0.492	0.903
Trend before intervention	Coefficients	0.149	0.052	0.123	0.131	0.196
	95% CI	0.011,0.287	−0.034,0.139	−0.063,0.309	−0.063,0.326	−0.060,0.452
	*P-value*	0.036	0.227	0.185	0.176	0.127
Level change after 1st intervention (week 6)	Coefficients	−0.759	−0.598	−0.506	−0.543	−0.957
	95% CI	−1.339,-0.178	−1.007,-0.190	−1.259,0.247	−1.330,0.243	−1.945,0.031
	*P-value*	0.013	0.006	0.178	0.167	0.057
Trend change after 1st intervention (week 6)	Coefficients	−0.204	−0.017	−0.125	−0.133	−0.191
	95% CI	−0.341,-0.067	−0.125,0.091	−0.309,0.060	−0.325,0.060	−0.449,0.067
	*P-value*	0.005	0.751	0.175	0.167	0.140
Level change after 2nd intervention (week 17)	Coefficients	0.070	−0.078	0.043	0.058	0.080
	95% CI	−0.029,0.168	−0.641,0.486	−0.035,0.122	−0.028,0.144	−0.047,0.206
	*P-value*	0.156	0.779	0.265	0.175	0.205
Trend change after 2nd intervention (week 17)	Coefficients	0.078	−0.032	0.000	−0.000	−0.008
	95% CI	0.064,0.092	−0.107,0.043	−0.008,0.009	−0.010,0.009	−0.020,0.004
	*P-value*	0.0001	0.392	0.980	0.918	0.161

*Note*: This table presents the results of single interrupted time series analyses between January 2020 and July 2020. The defendant variable is the change in the volume of patients per week

### Multiple group ITSA

**Fig. 2:**
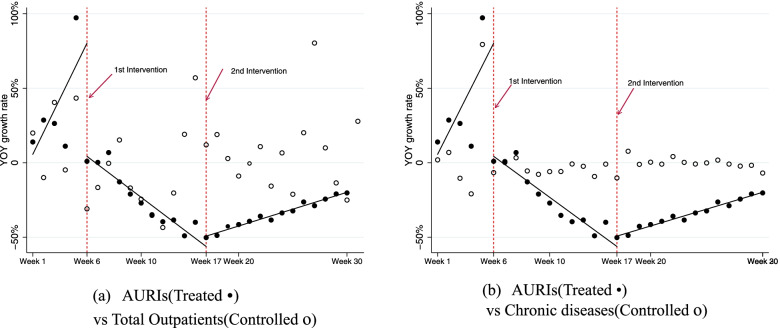
Presentation of multiple group ITSA with two interventions for AURIs versus chronic diseases. *Note*: Fig. 2 represents the multiple groups interrupted time series analysis with two interventions. The treated group is AURIs while total outpatients and chronic diseases (hypertension as well as diabetes) are identified as control group. The outcome measure is the yoy growth rate for the number of patients from each disease per week between January–July, 2017 and 2020. Black dot is the yoy of treated group while white dot as that of control group. The black line presents the trend of the yoy of treated group before and after the interventions

We use multiple group ITSA to assess the impact of the COVID-19 pandemic and social distancing in reducing healthcare utilisation for the elderly, using a multiple-group design. We compare patients from AURI’s diagnosis with those of the other diseases. Figure 2. presents the intervention effect for AURIs as the treatment group and that for other diseases as the control group. First, the upper panel shows the results of multiple group ITSA as AURIs for the treatment group versus total outpatients as the control group. Next, the lower panel shows the results of multiple group ITSA as AURIs for the treated versus chronic diseases as the control group. This represents the multiple group ITSA with two interventions. The treated group is AURIs, while total outpatients and chronic diseases (hypertension as well as diabetes) are identified as the control group. The outcome measure is the yoy growth rate for the number of patients from each disease per week between January–July, 2017 and 2020. The first intervention is week 6, 2020, and the second one is week 17, 2020.

Table 3 presents the multiple group ITSA results for the two intervention periods. For healthcare utilisation, AURIs cases are considered as the treatment group, and cases with other diseases are considered as the control group.

**Table 3:** Estimated results for multiple group ITSA with two interventions for AURIs versus chronic diseases

Treated: AURI		Total	Hypertension	Diabetes	Cancer
Intercept	Coefficients	0.004	−0.002	0.051	0.200
	95% CI	−0.105,0.113	−0.085,0.082	−0.040,0.142	0.035,0.364
	*P-value*	0.944	0.972	0.270	0.017
Trend before the 1st intervention (week 6)	Coefficients	0.000	0.000	0.000	0.001
	95% CI	−0.001,0.002	−0.001,0.002	−0.001,0.002	−0.001,0.004
	*P-value*	0.652	0.630	0.473	0.180
Level change after the 1st intervention (week 6)	Coefficients	−0.256	−0.075	−0.095	−0.314
	95% CI	−0.556,0.044	−0.172,0.022	−0.202,0.013	−0.471,-0.157
	*P-value*	0.094	0.128	0.086	<0.0001
Trend change after 1st intervention (week 6)	Coefficients	0.032	−0.004	−0.005	0.006
	95% CI	−0.040,0.103	−0.012,0.003	−0.013,0.004	−0.008,0.020
	*P-value*	0.383	0.231	0.297	0.392
The difference in level before the 1st intervention (week6)	Coefficients	0.023	0.028	−0.024	−0.173
	95% CI	−0.128,0.173	−0.105,0.161	−0.162,0.113	−0.368,0.021
	*P-value*	0.768	0.680	0.726	0.080
The difference in trend before the 1st intervention (week6)	Coefficients	0.000	0.000	0.000	−0.001
	95% CI	−0.002,0.003	−0.002,0.002	−0.002,0.002	−0.003,0.002
	*P-value*	0.832	0.758	0.905	0.540
The difference in level after the 1st intervention (week6)	Coefficients	0.163	−0.018	0.001	0.221
	95% CI	−0.162,0.487	−0.176,0.140	−0.164,0.167	0.020,0.422
	*P-value*	0.325	0.822	0.988	0.031
The difference in trend after the 1st intervention (week6)	Coefficients	−0.090	−0.053	−0.053	−0.064
	95% CI	−0.163,-0.016	−0.071,-0.036	−0.071,-0.036	−0.085,-0.043
	*P-value*	0.017	<0.0001	<0.0001	<0.0001
Level change after the 2nd intervention (week 17)	Coefficients	−0.066	0.053	0.069	0.070
	95% CI	−0.601,0.470	−0.020,0.126	−0.012,0.149	−0.045,0.185
	*P-value*	0.810	0.157	0.096	0.231
Trend change after the 2nd intervention (week 17)	Coefficients	−0.029	0.002	0.002	−0.011
	95% CI	−0.107,0.049	−0.005,0.010	−0.007,0.011	−0.024,0.003
	*P-value*	0.468	0.530	0.638	0.122
The difference in level after the 2nd intervention (week 17)	Coefficients	0.144	0.026	0.010	0.009
	95% CI	−0.400,0.688	−0.097,0.148	−0.118,0.137	−0.143,0.160
	*P-value*	0.603	0.682	0.879	0.911
The difference in trend after the 2nd intervention (week 17)	Coefficients	0.109	0.077	0.078	0.091
	95% CI	0.029,0.188	0.060,0.095	0.060,0.095	0.070,0.111
	*P-value*	0.008	<0.0001	<0.0001	<0.0001

*Note*: This table presents the results of multiple-group interrupted time series analyses between January – July from 2017–2020. The treated group is AURIs, while control groups include total ambulatory services, chronic diseases, and cancer (only including patients taking ambulatory services)

As shown in Table 3, the initial mean level difference of the yoy growth rate between AURIs and both chronic diseases are not statistically significant (*p* = 0.966, confidence interval [CI] -0.119 to 0.124). Moreover, the difference in the mean baseline slope is not distinctive either (*p* = 0.758, CI −0.002 to 0.002). Hence, chronic diseases are comparable with AURIs on baseline level and trend before the intervention. There is no statistically significant treatment effect of the first intervention of week 6, whereas there is a statistically significant decline in the post trend compared with that of chronic diseases groups of 5.3% (*p* < 0.001). Next, as the social distancing measures were lifted at the second intervention of week 17, the healthcare utilisation for AURIs turns upwards by 7.8% (*p* < 0.001) compared with hospital visits for chronic diseases.

## Discussion

### Main findings of this study

From the start of the COVID-19 pandemic to the recovery period in the first half of 2020 in South Korea, there was no significant change in the outpatient utilisation of medical facilities for hypertension, diabetes, or cancer among elderly people, while the outpatient visits for AURIs noticeably decreased. Our findings suggest that the COVID-19 pandemic has not kept elderly people from accessing essential medical services in South Korea. These encouraging results may be partly due to the characteristics of South Korea’s appropriately mixed social distancing policies, i.e., the physical distance between people is voluntarily maintained, but formal control over movement is minimised at the same time, the appropriate supply of hospital services provided to non-COVID-19 patients. Since January 20, 2020, when South Korea’s first known case of COVID-19 was identified, the government has educated the public using a variety of media platforms on the importance of personal hygiene, such as wearing masks and handwashing, and there is widespread compliance with the recommended measures. The public healthcare system used a test-and-trace strategy appropriately mixed with social distancing policies.^2^ Hospitals provided the logistics for isolation and intensive care treatment for COVID-19 patients and medical care for non-COVID-19 patients at the same time. COVID-19 patients were quarantined in medical facilities designated by the government while receiving medical treatment. Non-COVID-19 patients used hospital care without disruption at regular medical institutions where infection prevention policies were applied. Hospitals adopted preventive procedures and adjusted the physical environments to minimise the risks of COVID-19 transmission within the healthcare setting, in accordance with the guidelines^3^ jointly announced by the government and the Society for Infection Control.^4^ The public was informed of the National Relief Hospital,^5^ appointed to ensure that the infection control measures were strictly kept, helping to relieve the anxiety of COVID-19 transmission in the hospital. In addition, temporary telehealth consultations were allowed, as well as proxy prescriptions, and dedicated respiratory clinics were endorsed for non-COVID-19 related health care utilisation [26]. Our findings support that a well-organized medical provision system can help patients access medical care even in pandemic situations [42].

### What is already known on this topic

A reduction in the outpatient utilisation of medical facilities for AURIs is consistent with the results of previous studies [43, 44]. First, restriction of medical resources due to sudden patient concentration or blockade of medical institutions affects the decline in the use of all diseases [1, 3]. Second, the incidence of respiratory infections could be reduced significantly through improved personal hygiene management and social distancing measures, including refraining from going out, closing schools, and working from home [45–47]. Immediately after the first COVID-19 outbreak, the South Korean government strongly urged people to practice personal protective measures, and people agreed to adhere to the hygiene guidelines. People voluntarily abstained from going out due to concerns about infection even before the implementation of the national social distancing measures, confirmed by the reduced public transport use and traffic volume at that time in South Korea [48, 49]. Third, people may have avoided hospital visits, despite symptoms resulting from AURIs and other diseases, due to the fear of acquiring COVID-19 infection [50].

### What this study adds

In South Korea, the overall number of visits associated with hypertension, diabetes, and cancer in the elderly population was similar to that in the past, even when the uncertainty and fear of infection were greatest. These results are contrasting to previous studies showing that healthcare utilisation related to these diseases decreased during COVID-19 because, in many countries, the use of essential medical services also decreased due to deteriorated functioning of medical institutions [5, 16, 17]. However, the results of previous studies that the healthcare utilisation gradually recovered from patients with hypertension and diabetes after a strong decrease in the initial stage are supported [1, 3].

### Robustness of the results

As discussed in Methods section, our main analysis has been carried out for the elderly group to those aged 65–84 years. To see the robustness of the results and gauge the influence of the age group, we also conduct an additional analysis for those aged 85 and older. We find that the results remain qualitatively intact: Specifically, the utilisation rate for chronic diseases changed little whereas for AURIs it dropped by 26.4% year-over-year (yoy) at the onset of the pandemic (week 6, 2020). Furthermore, as social distancing relaxed (week 17, 2020), the AURIs patients trended up and even reached to 6.95% above yoy whereas no significant change found for chronic diseases, which largely supports the implications drawn from our main analysis.^6^

### Limitations of this study

There are some limitations to interpreting the present study results. First, the observation period of the study was in the early stage of the COVID-19 pandemic in South Korea. This was a time when the threat of infectious disease transmission was very high due to the fear of COVID-19 and the uncertain policy response. Due to the surge of COVID-19 infection among the religious group in the Daegu and Gyeongbuk area, there may be some limitations to interpreting nationwide medical utilisation. In the future, it will be necessary to analyse the difference in medical visits between regions for a more accurate interpretation. Second, in this study, representative chronic and acute diseases necessitating frequent outpatient visits were selected. To determine changes in healthcare utilisation, a more specific level of medical treatment data should be selected. However, to comprehensively interpret the impact on the South Korean medical system during the COVID-19 pandemic, additional analysis on hospitalisation, emergency, and severe medical care will be required. Third, the estimate of effect size in ITSA is dependent on the intervention timing. We deliberately chose the first intervention date after the week of the lunar new year holiday, which might affect the hospital visits, but before the official social distancing policy was enforced. There were four weeks of delays in putting mandated social distancing measures after the first COVID-19 case. Finally, we only chose ‘hypertension’ and ‘diabetes’, representing chronic diseases; both hypertension and diabetes among chronic diseases, are difficult to recognise the symptoms immediately even if the management is delayed a little. It is relatively likely to delay treatment in situations during the COVID-19 pandemic.^7^ Furthermore, from the early period of the COVID-19 pandemic, hypertension and diabetes were known to have a positive association with the severity of COVID-19, raising fears about their infection and increasing the possibility of deferring or giving up hospital visits. Moreover, some antihypertensive drugs have been reported to increase susceptibility to COVID-19, mounting the likelihood of non-compliance [51–53].

## Conclusions

We analysed the yoy growth in the number of patients for AURIs and chronic diseases among elderly people to investigate the healthcare utilisation in South Korea during the COVID-19 pandemic. The patients associated with AURIs showed large decreases, while visits for chronic diseases were similar to pre-COVID-19 trends. Our findings imply that the South Korean public health authority effectively managed the medical system to accommodate patients who need regular check-ups and treatment along with a strong COVID-19 containment policy in the healthcare settings, and its dual strategy works to maintain the healthcare system even in the middle of an infectious disease crisis.

Furthermore, our findings emphasise that with the ongoing pandemic, it is important to monitor the health effects of non-COVID-19 patients, particularly those who have chronic diseases and those deferring hospital visits. In addition, older patients with existing health conditions should be aware of the importance of their hospital visits, compliance, and regular check-up about their health conditions. Suspending the diagnosis of chronic diseases for older patients can lead to adverse health consequences and higher mortality. Therefore, it is the utmost challenge for public healthcare authorities to keep providing timely medical care to non-COVID-19 patients; furthermore, patients with existing health concerns should be motivated with their healthcare problems until the COVID-19 pandemic ends.

## Supplementary Information

**Additional file 1: Table S1.** Estimated regression results of single ITSA (cardiovascular diseases and stroke). **Table S2.** Estimated regression results of single ITSA (dementia and musculoskeletal disorder). **Figure S1.** Graphical Representation of Single ITSA (Cardiovascular diseases, Stroke, Dementia, and Musculoskeletal disorders) . **Table S3.** Estimated regression results of single ITSA for the elderly aged 85 and older. **Figure S2.** Presentation of single ITSA for the elderly aged 85 and older (chronic diseases, AURIs, and influenza).

## Data Availability

The data used in this study were provided by National Health Insurance Service Korea. Data will be shared on request to the corresponding author with the permission of the National Health Insurance Service Korea.

## Footnotes

1 Just to note, however, our main results remain qualitatively intact even for the very olds aged 85 and older. We relegate the related results separately as supplemental materials (see Table S3 and Figure S2 in Supplement).

2 Ministry of Health and Welfare, Korea Center for Disease Control and Prevention (2020.2.) at the following link: http://ncov.mohw.go.kr/socdisBoardView.do?brdId=6&brdGubun=1&dataGubun=&ncvContSeq=&contSeq=&board_id=&gubun

3 Central Defence Countermeasure Headquarters. (2020.4.) Prevention and management of coronavirus infection-19 infection in medical institutions (for nursing hospitals)

4 Korea Centers for Disease Control and Prevention, Korean Society for Medical Infection Control, Korean Society for Infection Control and Nurses, Korean Society for Infectious Diseases (2020.2.) Preventing and managing new coronavirus infections (for clinic-level medical institutions)

5 Ministry of Health and Welfare press release (February 24, 2020). Operation of ‘National Relief Hospital’ that is safe from COVID-19 infection.

6 For further information, refer to Table S3 and Figure S2 from Supplement.

7 We extend our analysis to other chronic diseases (i.e., cardiovascular diseases, stroke, dementia and musculoskeletal disorder). The results are broadly consistent with our original analysis; the decreasing trend of HC at the first intervention (6th week in 2020) is emerged for most of diseases. However, any changes at the 2nd intervention (17th week in 2020) were not clear (refer to Table S1, S2, and Figure S1 in Supplement).
