# Supplementary material for: Healthcare utilisation for elderly people at the onset of the COVID-19 pandemic in South Korea
Source: BMC Geriatr. 2022 May 6;22:395. doi: 10.1186/s12877-022-03085-5 (PMC9072758; doi:10.1186/s12877-022-03085-5)
Supplement: Supplementary file 1 — Additional file 1: Table S1. Estimated regression results of single ITSA (cardiovascular diseases and stroke). Table S2. Estimated regression results of single ITSA (dementia and musculoskeletal disorder). Figure S1. Graphical Representation of Single ITSA (Cardiovascular diseases, Stroke, Dementia, and Musculoskeletal disorders) . Table S3. Estimated regression results of single ITSA for the elderly aged 85 and older. Figure S2. Presentation of single ITSA for the elderly aged 85 and older (chronic diseases, AURIs, and influenza). [file 12877_2022_3085_MOESM1_ESM.docx]

**Supplement**

**Table S1.** Estimated regression results of single ITSA (cardiovascular diseases and stroke)^[[1]](#footnote-1)^

|  | Cardiovascular D | | Stroke | |
| --- | --- | --- | --- | --- |
|  | visit | patient | visit | patient |
| Trend before intervention | 0.2059** | 0.2870*** | 0.0974 | 0.2363** |
|  | (0.073) | (0.072) | (0.122) | (0.071) |
| Level change after 1^st^ intervention (week 6) | -0.9117** | -1.0058** | -0.5988 | -0.8455** |
|  | (0.322) | (0.308) | (0.464) | (0.298) |
| Trend change after 1^st^ intervention (week 6) | -0.2047** | -0.2883*** | -0.1033 | -0.2389** |
|  | (0.072) | (0.071) | (0.123) | (0.071) |
| Trend change after 1^st^ intervention (week 6) | 0.1110 | 0.0222 | 0.1243 | 0.0191 |
|  | (0.096) | (0.101) | (0.132) | (0.066) |
| Trend change after 2^nd^ intervention (week 17) | 0.0049 | 0.0082 | 0.0103 | 0.0078 |
|  | (0.010) | (0.010) | (0.013) | (0.006) |
| Intercept | -0.2513* | -0.4722*** | 0.1103 | -0.4183*** |
|  | (0.110) | (0.126) | (0.218) | (0.113) |
| N | 53 | 53 | 53 | 53 |
| Note: This table presents the results of single interrupted time series analyses. The first intervention is 6^th^ week and the second intervention 17^th^ week, 2020. The defendant variable is the change in the volume of visits and patients per week from the previous year (yoy). The parenthesis is Newey-West standard error and the significance levels are * 0.05 ** 0.01 ***0 .001. | | | | |

**Table S2.** Estimated regression results of single ITSA (dementia and musculoskeletal disorder)

|  | Dementia | | Musculoskeletal disorder | |
| --- | --- | --- | --- | --- |
|  | visit | patient | visit | patient |
| Trend before intervention | 0.2615* | 0.2111*** | 0.1489** | 0.1794*** |
|  | (0.111) | (0.059) | (0.053) | (0.051) |
| Level change after 1^st^ intervention (week 6) | -1.0314* | -0.6866** | -0.6594** | -0.7124** |
|  | (0.453) | (0.239) | (0.229) | (0.206) |
| Trend change after 1^st^ intervention (week 6) | -0.2706* | -0.2218*** | -0.1670** | -0.1867*** |
|  | (0.112) | (0.059) | (0.055) | (0.052) |
| Trend change after 1^st^ intervention (week 6) | 0.1561 | 0.0378 | 0.2075 | 0.1134 |
|  | (0.086) | (0.072) | (0.106) | (0.066) |
| Trend change after 2^nd^ intervention (week 17) | 0.0154 | 0.0191** | 0.0237 | 0.0120 |
|  | (0.009) | (0.007) | (0.014) | (0.010) |
| Intercept | -0.3732* | -0.3838** | -0.1516 | -0.3002** |
|  | (0.165) | (0.115) | (0.096) | (0.111) |
| N | 53 | 53 | 53 | 53 |
| Note: This table presents the results of single interrupted time series analyses. The first intervention is 6^th^ week and the second intervention 17^th^ week, 2020. The defendant variable is the change in the volume of visits and patients per week from the previous year (yoy). The parenthesis is Newey-West standard error and the significance levels are * 0.05 ** 0.01 ***0 .001. | | | | |

**Figure S1.** Graphical Representation of Single ITSA ( Cardiovascular diseases, Stroke, Dementia, and Musculoskeletal disorders)

| 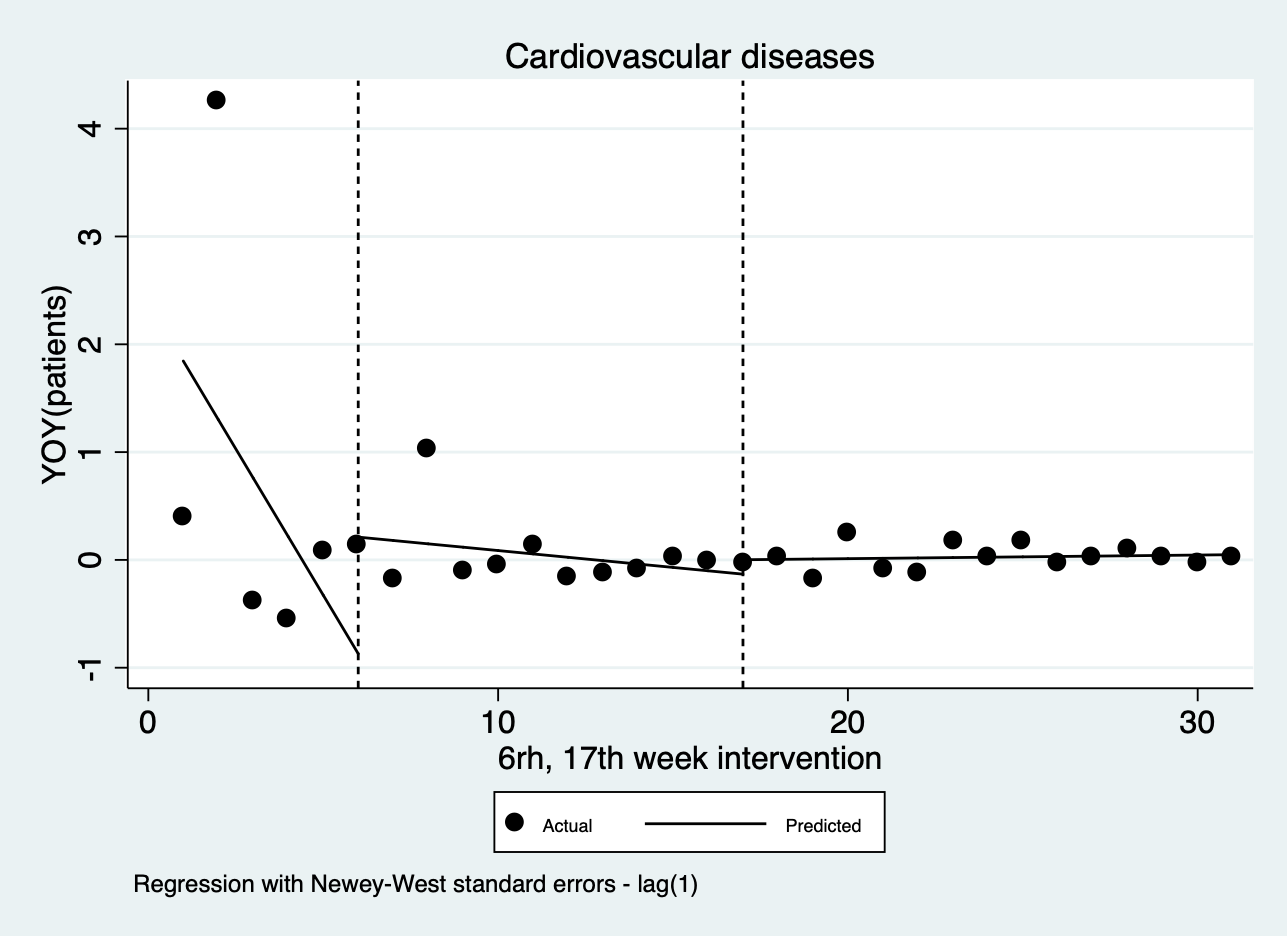 |
| --- |
| (a) Cardiovascular diseases |
| 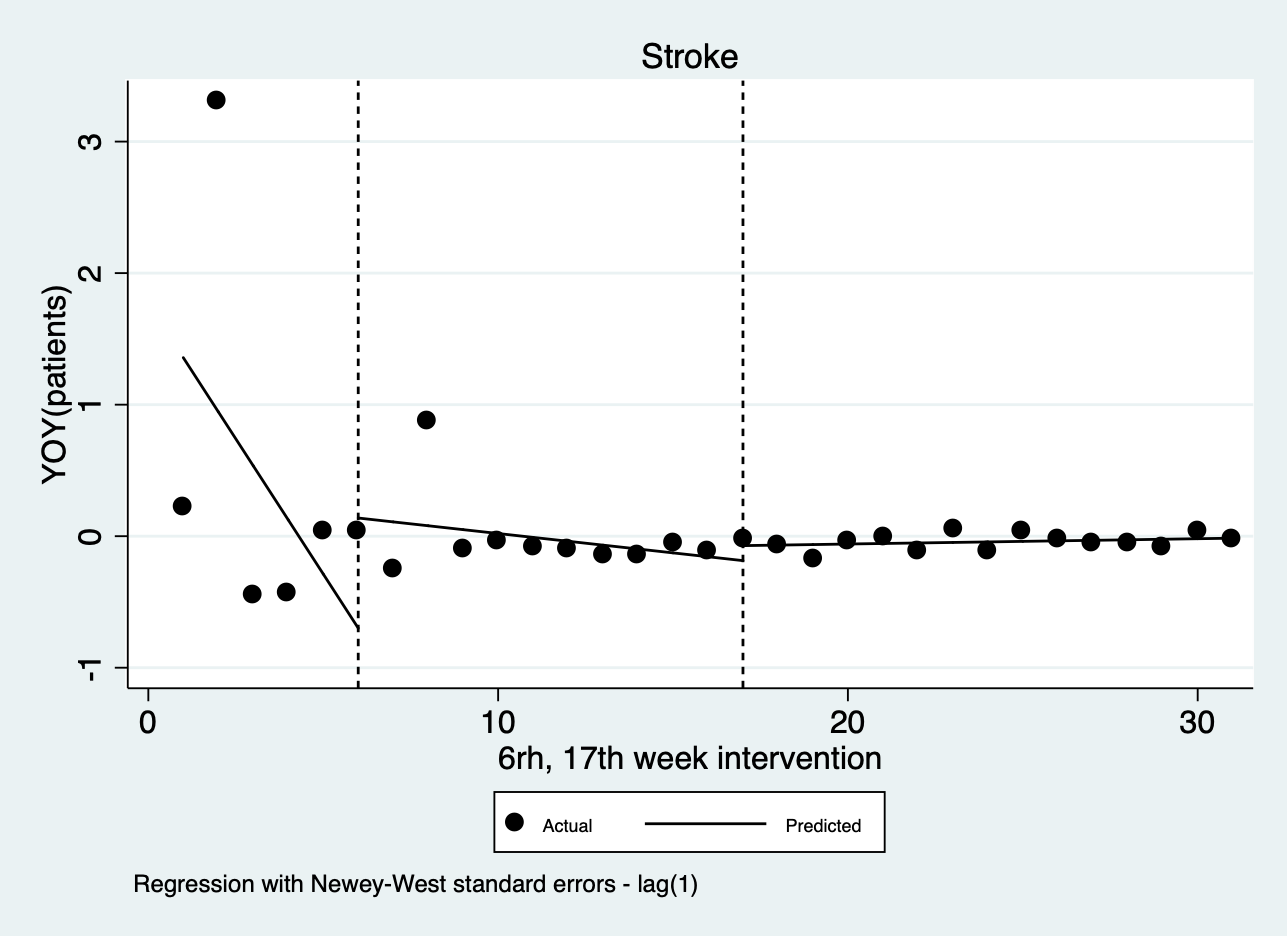 |
| (b) Stroke |

Note: Figure S1 represents the single interrupted time series analysis with two interventions.

The outcome measure is the YOY growth rate for the number of patients from each disease per week

from the previous year. The first intervention is week 6, 2020 and the second one week 17, 2020.

| 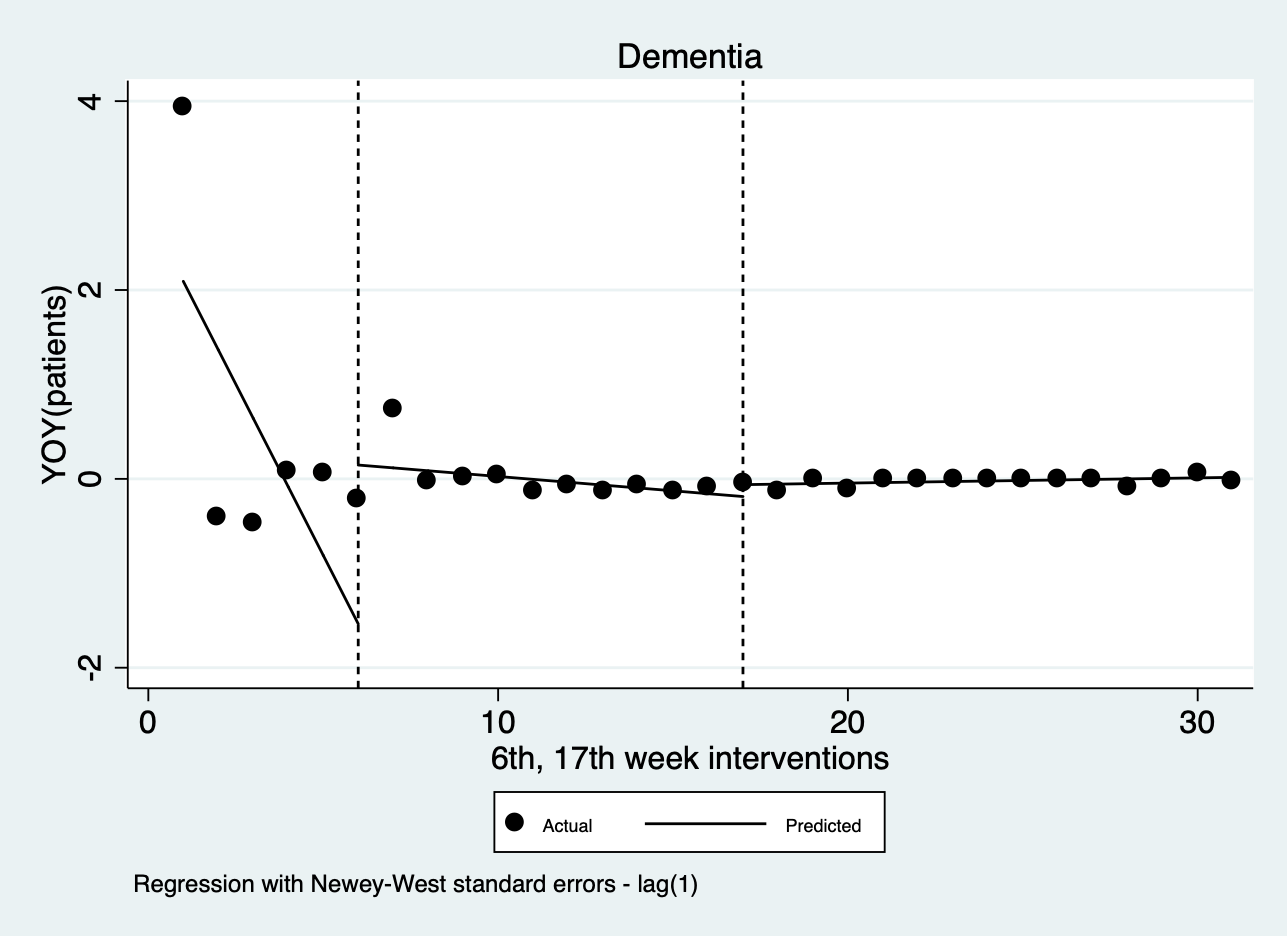 |
| --- |
| (c) Dementia |
| 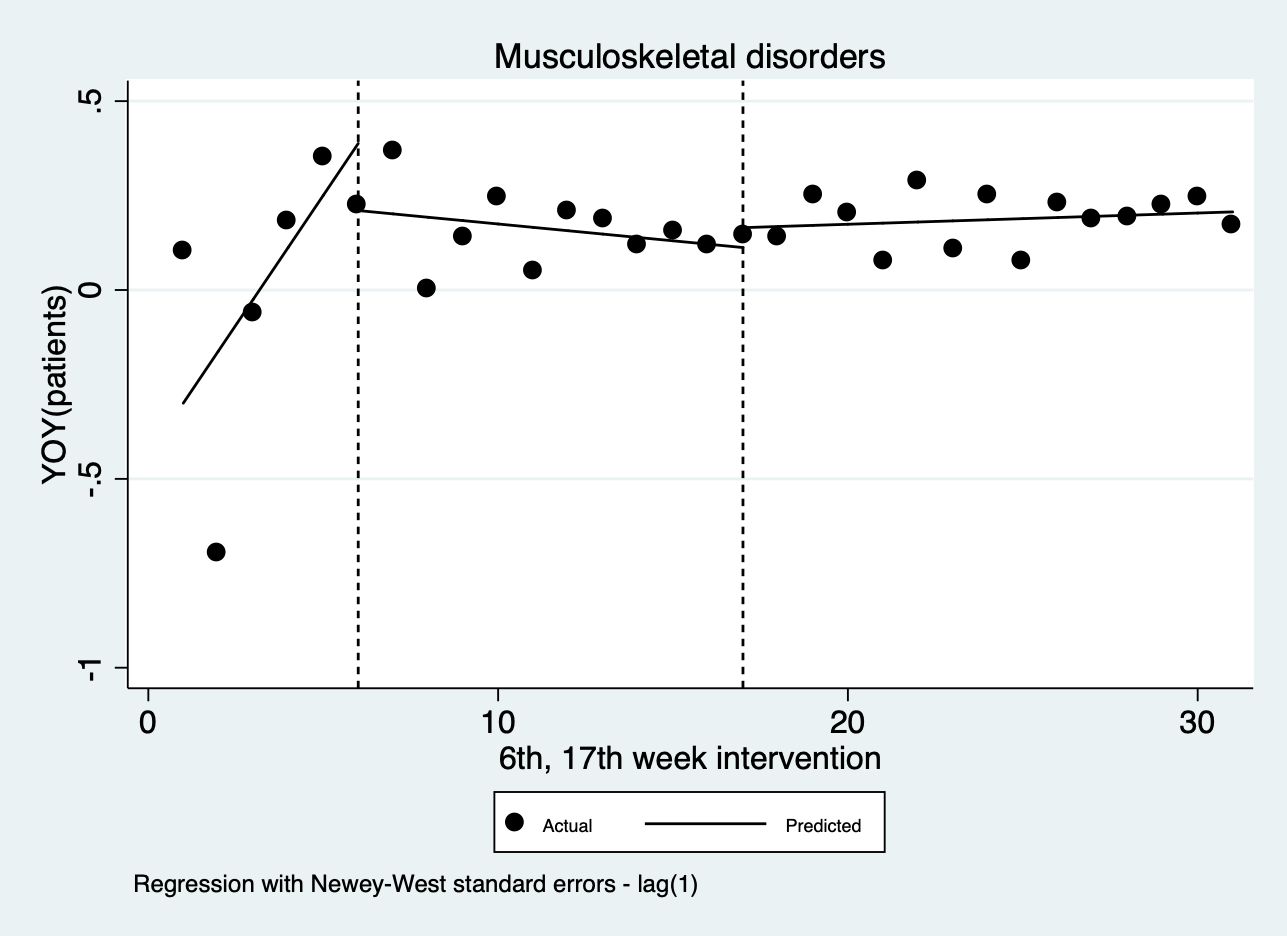 |
| (d) Musculoskeletal disorders |

**Table S3.** Estimated regression results of single ITSA for the elderly aged 85 and older

|  | (1) | (2) | (3) | (4) |
| --- | --- | --- | --- | --- |
|  | Hypertension | Diabetes | AURIs | Influenza |
| Trend before intervention | 0.1480 | 0.1490 | 0.2137* | 0.6975*** |
|  | (0.105) | (0.107) | (0.098) | (0.044) |
| Level change after 1^st^ intervention (week 6) | -0.6216 | -0.6507 | -0.9033* | -3.5737*** |
|  | (0.423) | (0.429) | (0.393) | (0.277) |
| Trend change after 1^st^ intervention (week 6) | -0.1497 | -0.1508 | -0.2640* | -0.8394*** |
|  | (0.104) | (0.107) | (0.099) | (0.043) |
| Trend change after 1^st^ intervention (week 6) | 0.0907 | 0.1020 | 0.0582 | 0.5489** |
|  | (0.076) | (0.060) | (0.050) | (0.194) |
| Trend change after 2^nd^ intervention (week 17) | -0.0088 | -0.0054 | 0.0695*** | 0.2011*** |
|  | (0.011) | (0.007) | (0.006) | (0.027) |
| Intercept | -0.1017 | -0.0290 | 0.0382 | 0.4169*** |
|  | (0.185) | (0.189) | (0.161) | (0.094) |
| N | 30 | 30 | 30 | 30 |

Note: This table presents the results of single interrupted time series analyses for patients older than 85 years. The first intervention is 6^th^ week and the second intervention 17^th^ week, 2020. The defendant variable is the change in the volume of patients per week from the previous year (yoy). The parenthesis is Newey-West standard error and the significance levels are * 0.05 ** 0.01 ***0 .001.

**Figure S2.** Presentation of single ITSA for the elderly aged 85 and older (chronic diseases, AURIs, and influenza)

| 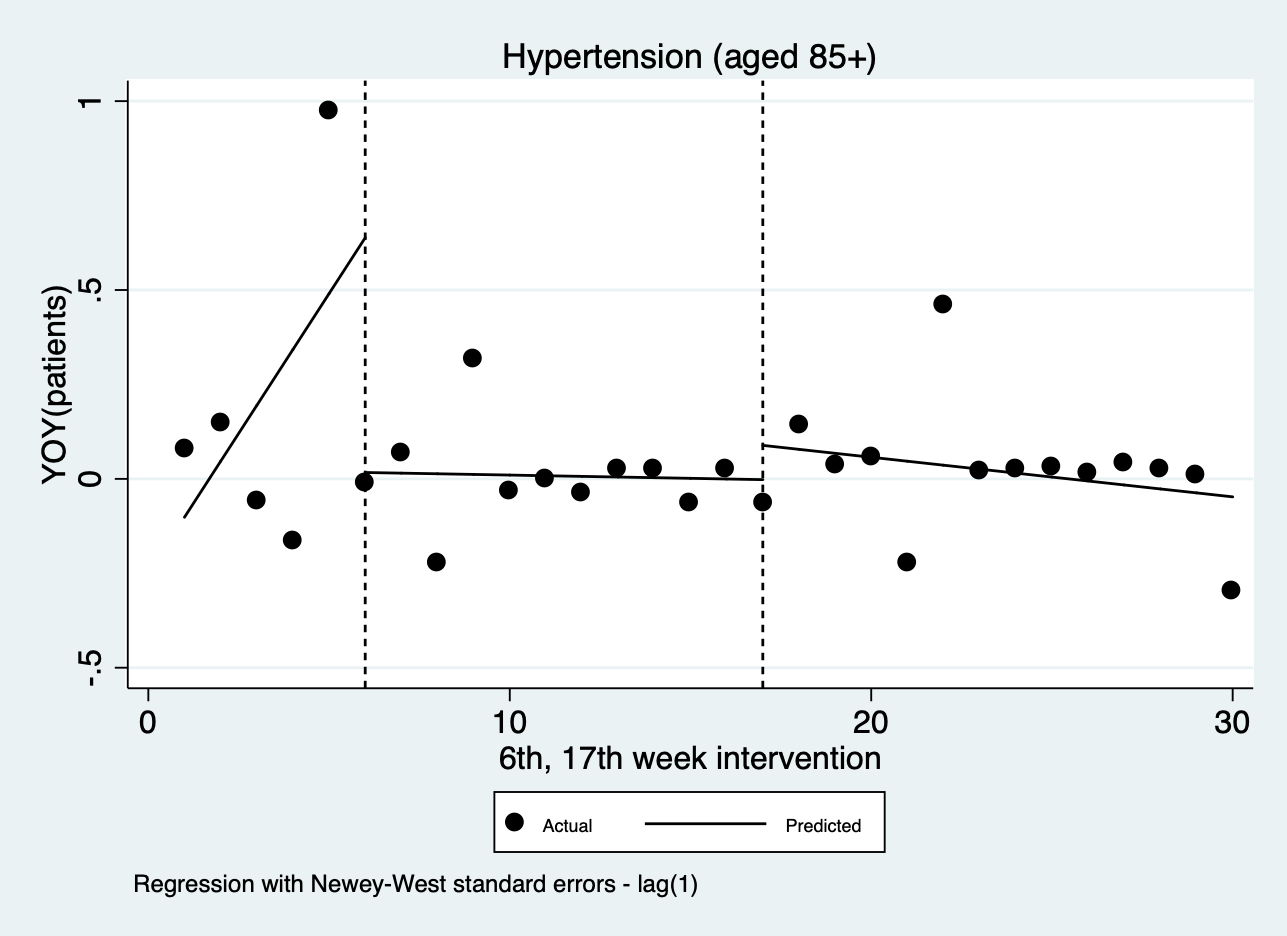 |
| --- |
| (a) Hypertension |
| 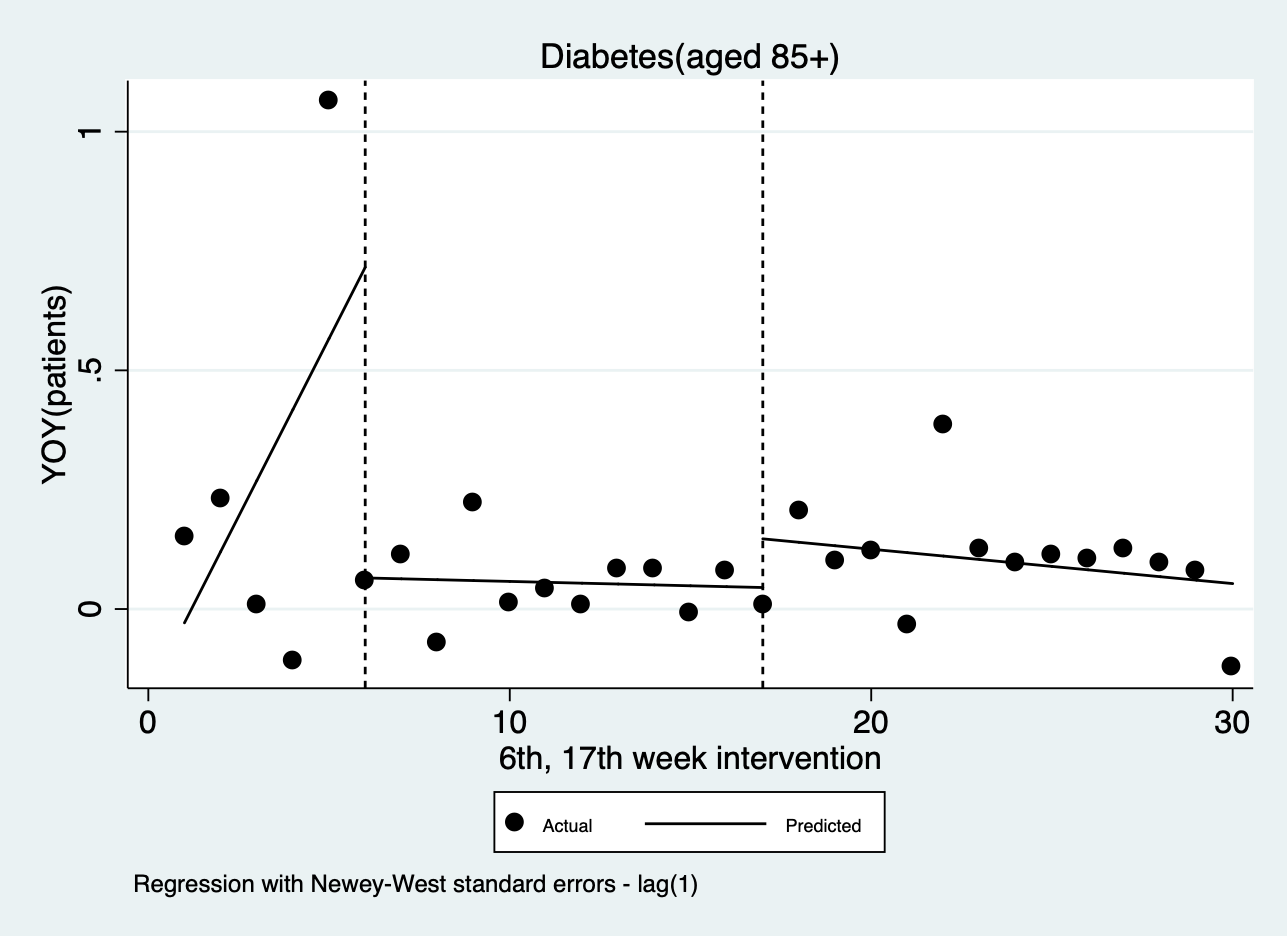 |
| (b) Diabetes |

| 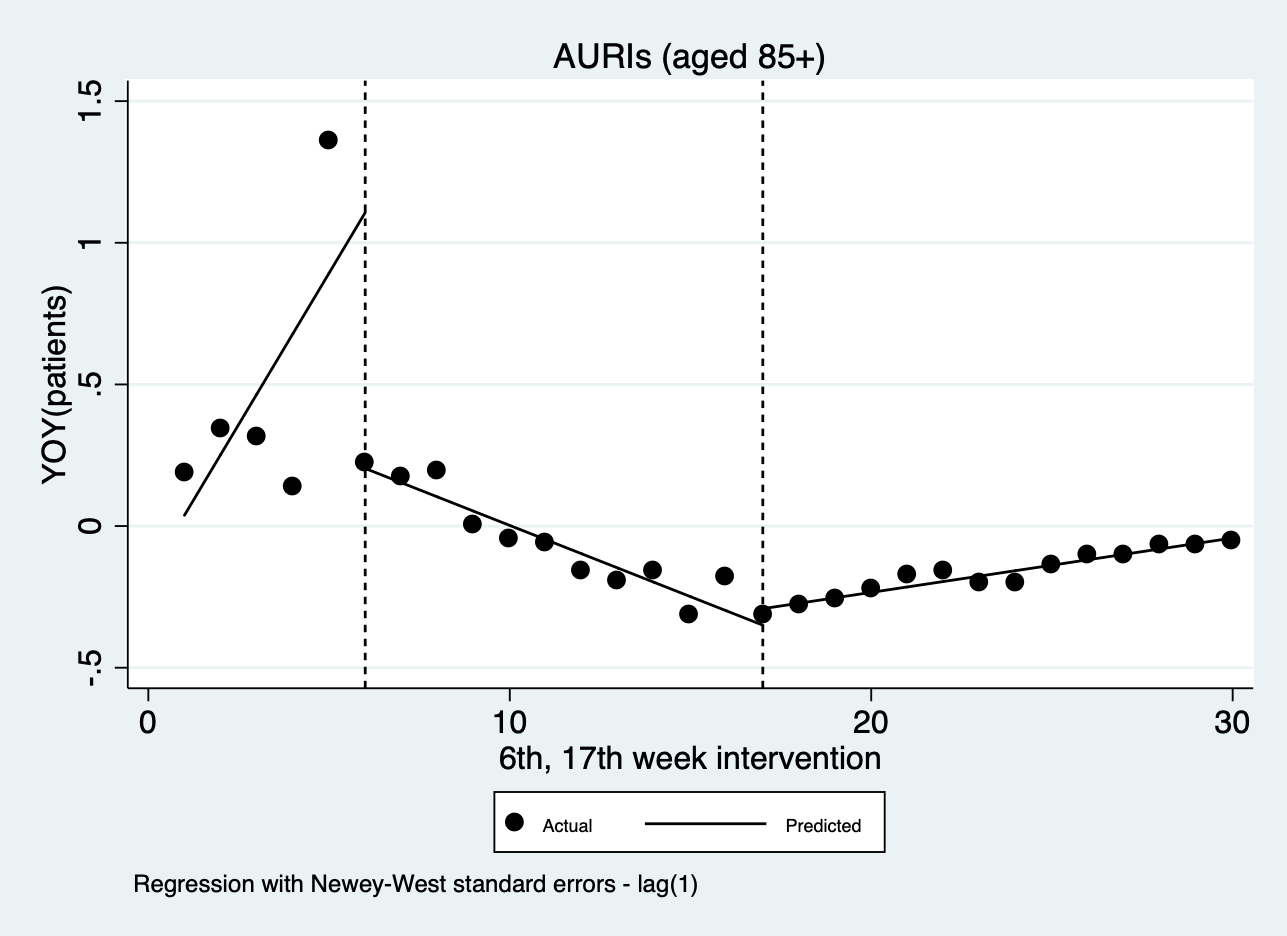 |
| --- |
| (c) AURIs |
| 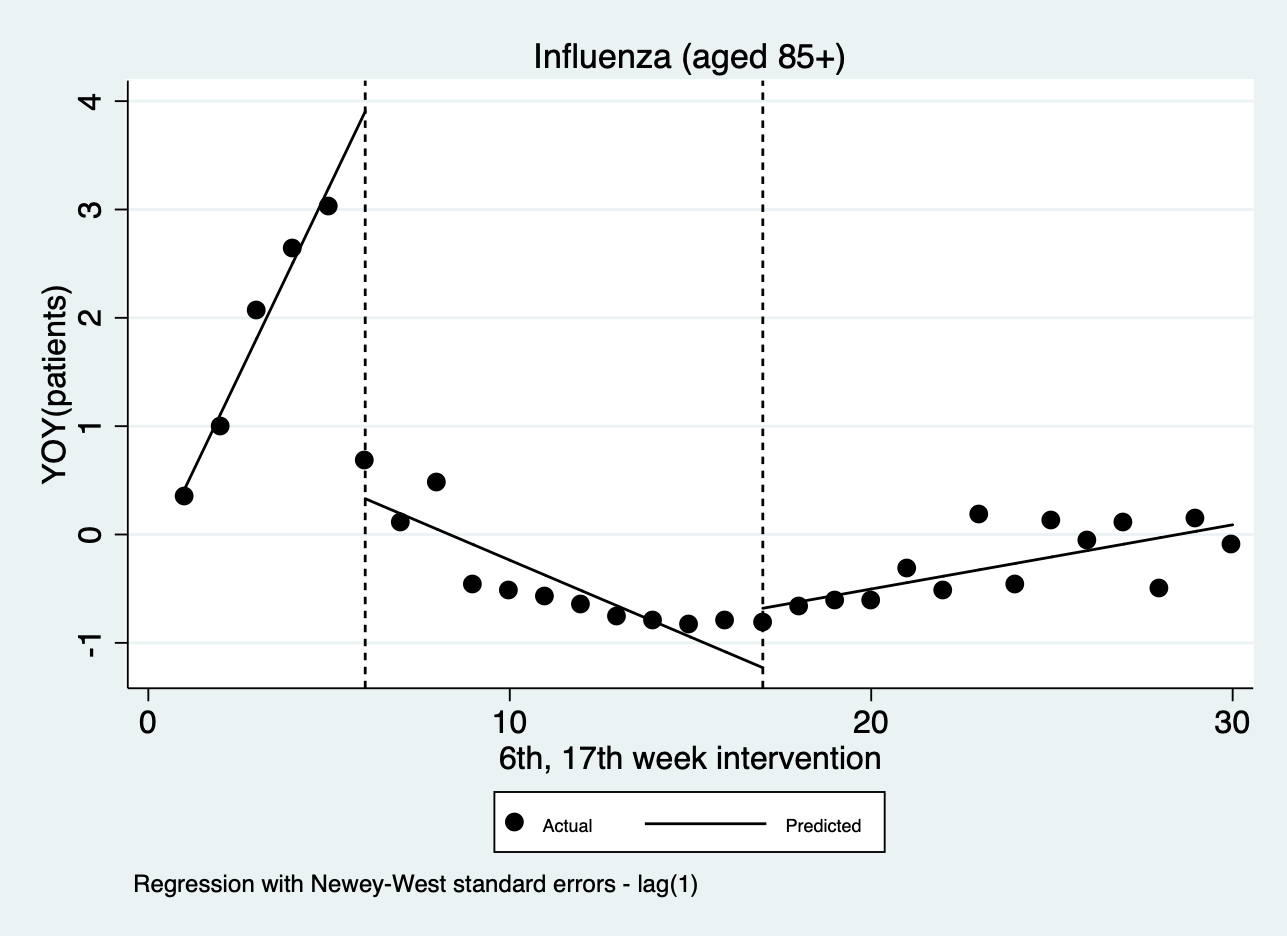 |
| (d) Influenza |

1. We identified the patient visit records pertaining to cardiovascular diseases according to the code I25(Chronic ischemic heart disease), from the International Statistical Classification of Diseases and Related Health Problems (ICD). We considered Strokes (ICD-10 codes: I63) and Dementia (ICD-10 codes: F00, F01, F02). Lastly, we use the data regarding Musculoskeletal disorder (ICD-10 codes: M91, M92, M93, M94, M95, M96). [↑](#footnote-ref-1)
